# Improved Cholesteatoma Removal with CADISS: A Quantitative Ultrastructural Comparison Using VP-SEM

**DOI:** 10.3390/jcm14124192

**Published:** 2025-06-12

**Authors:** Michela Relucenti, Ubaldo Romeo Plastina, Pasquale Fino, Chiara Filippi, Maurizio Barbara, Edoardo Covelli

**Affiliations:** 1 Department of Anatomy, Histology, Forensic Medicine and Orthopedics, Sapienza University of Rome, Via Alfonso Borelli 50, 00161 Rome, Italy; ubaldo.romeoplastina@uniroma1.it; 2 Department of Clinical Internal, Anesthesiological and Cardiovascular Sciences, Dermatology Clinic, University of Rome “La Sapienza”, 00185 Rome, Italy; pasquale.fino@gmail.com; 3 Department of Neuroscience, Mental Health and Sensory Organs, Faculty of Medicine and Psychology, Sapienza University of Rome, 00161 Rome, Italy; chiara.filippi@uniroma1.it (C.F.); maurizio.barbara@uniroma1.it (M.B.); edoardo.covelli@uniroma1.it (E.C.)

**Keywords:** cholesteatoma surgery, chemical-assisted dissection, VP-SEM imaging, residual disease

## Abstract

**Background**: Cholesteatoma is a destructive middle ear pathology requiring precise surgical removal to prevent recurrence and preserve auditory function. The chemically assisted dissection (CADISS) system (AuXin Surgery, Ottignies-Louvain-la-Neuve, Belgium), based on Mesna (5%), was introduced to enhance tissue separation and minimize residual disease. **Objective**: This study aimed to compare the cleaning efficiency of CADISS-assisted dissection versus the conventional manual dissection of cholesteatoma from incus bone surfaces using quantitative ultrastructural analysis. **Methods**: This retrospective study evaluated 67 human incus samples collected during cholesteatoma surgery—35 treated with manual dissection and 32 with CADISS. Samples were imaged using variable pressure scanning electron microscopy (VP-SEM) in hydrated conditions. Clean area/total area ratios were calculated and analyzed statistically using non-parametric tests. Postoperative MRI follow-up at 1 month was conducted to assess residual disease. **Results**: CADISS-assisted samples demonstrated significantly higher clean area/total area ratios (mean: 0.2095 vs. 0.0478, *p* < 0.0001). Qualitative imaging showed fewer residuals > 1 mm in the CADISS group (9% vs. 77%). MRI follow-up revealed a lower recurrence rate in the CADISS group (3.1%) compared to manual dissection (11.4%). **Conclusions**: CADISS-assisted dissection provides superior cholesteatoma debris removal compared to manual methods, as evidenced by VP-SEM imaging and clinical follow-up. This technique may improve surgical outcomes and reduce recurrence risk in middle ear surgery.

## 1. Introduction

Cholesteatoma is a progressive, destructive disease characterized by the abnormal accumulation of keratinizing stratified squamous epithelium within the middle ear and mastoid cavity [1]. It frequently arises as a complication of chronic otitis media and is associated with severe consequences such as conductive hearing loss; ossicular chain erosion; and, in advanced cases, intracranial extension due to bone destruction [2,3]. Cholesteatomas are typically classified as either congenital or acquired. The acquired form, which is more prevalent, is often associated with Eustachian tube dysfunction, leading to tympanic membrane retraction and keratin debris entrapment [1]. In contrast, congenital cholesteatomas are believed to arise from ectopic epithelial remnants and present behind an intact tympanic membrane without prior infections [4]. Surgical removal remains the cornerstone of cholesteatoma treatment, with the primary objective being complete disease eradication while preserving hearing. Traditional microscopic techniques, such as atticotomy and mastoidectomy, are typically performed using either canal wall-up or canal wall-down approaches, each offering specific advantages and trade-offs [5,6]. In recent years, transcanal endoscopic ear surgery (TEES) has gained traction as a minimally invasive alternative, enhancing the visualization of hidden recesses like the sinus tympani and facial recess [5,7,8,9].

Advancements in surgical technology, including ultrasonic dissection tools and augmented reality-guided navigation, have significantly enhanced the precision of cholesteatoma removal [7,10]. Furthermore, combined microscopic–endoscopic approaches have been explored to improve visualization in complex cases, particularly when the disease extends into hard-to-reach areas, such as the petrous apex [11,12].

One notable innovation is the introduction of chemically assisted dissection, particularly with Mesna-based agents such as the CADISS^®^ system. Mesna (sodium 2-mercaptoethanesulfonate, 5%) functions by breaking disulfide bonds within keratinized tissues, allowing for selective loosening of the cholesteatoma matrix from healthy bone surfaces [13]. This technique may help reduce residual disease and surgical trauma, particularly in anatomically challenging regions [14,15]. The use of Mesna (sodium 2-mercaptoethanesulfonate) as a surgical adjunct has gained increasing attention due to its mucolytic and tissue-sparing properties, particularly in otolaryngology, orthopedics, and gynecology. Mesna acts by cleaving disulfide bonds within extracellular matrix proteins, thereby weakening tissue adhesions and facilitating dissection along natural cleavage planes. This mechanism is especially advantageous in conditions such as cholesteatoma and adhesive otitis media, where pathological tissues adhere tightly to delicate structures [13,14].

Several studies have confirmed the clinical utility of Mesna in enhancing both the safety and efficiency of surgical procedures. Mesna significantly improves the management of atelectatic ears and adhesive otitis media by enabling safer and more effective elevation of the tympanic membrane [15]. Dissection in proximity to critical neurovascular structures, such as the sigmoid sinus and facial nerve, is facilitated by Mesna without inducing systemic toxicity or local neurotoxicity [16].

Beyond its mechanical and chemical dissection benefits, Mesna has also been associated with potential antimicrobial and protective roles, and its use may contribute to reduced surgical site infections, suggesting broader perioperative benefits that extend beyond tissue separation [17].

The advantages of Mesna are not limited to otologic surgery. In spine surgery, particularly in revision lumbar discectomies, and in gynecological procedures such as myomectomy, Mesna has been shown to reduce dissection time and postoperative complications [18,19,20].

Despite these advancements, the most effective surgical approach remains debated and often must be tailored to individual anatomical and clinical contexts. This study aimed to quantitatively compare manual dissection with CADISS-assisted dissection in cholesteatoma surgery, using variable pressure scanning electron microscopy (VP-SEM) to evaluate cleaning efficiency on incus bone samples. Postoperative MRI was also used to assess the presence of residual disease, allowing for a comprehensive comparison between these two techniques.

## 2. Materials and Methods

### 2.1. Study Design and Objectives

This retrospective study aimed to compare the cleaning efficiency of CADISS-assisted dissection versus manual dissection in adult cholesteatoma surgery. The evaluation focused on the removal of residual cholesteatoma from incus bone surfaces using ultrastructural imaging. A total of 67 incus samples were analyzed: of those, 35 underwent manual dissection, and 32 were treated with the CADISS-assisted technique. The primary endpoint was the clean area/total area ratio, calculated using images acquired by variable pressure scanning electron microscopy (by VP-SEM Hitachi 3500, Hitachi, Japan). For each sample, 10 images were evaluated, yielding 350 measurements in the manual group and 320 in the CADISS group.

### 2.2. Sample Collection and Grouping

Samples were collected intraoperatively from 67 patients aged between 18 and 85 years undergoing cholesteatoma surgery. Based on the dissection technique employed, samples were divided into two groups: incus bones subjected to standard mechanical removal of cholesteatoma using microsurgical tools were designated as the manual dissection group (n = 35). Incus bones subjected to chemically assisted dissection using the CADISS^®^ system (Mesna 5%), followed by gentle mechanical dissections, were categorized as the CADISS group (n = 32).

### 2.3. Inclusion and Exclusion Criteria and Study Approval

Patients undergoing surgery for cholesteatoma with visible residual tissue on the incus bone samples at the time of extraction were included in this study. Patients with the presence of unrelated pathological bone conditions or incus bones that had been damaged during surgical removal were excluded from the study. The study protocol received approval from the Institutional Review Board (Protocol No. 0862/2023, Ref. 7143, approval date 18 October 2023), ensuring adherence to ethical guidelines for research involving human tissues.

### 2.4. Surgical Procedures

All procedures were performed by Prof. Maurizio Barbara, a senior otologic surgeon with 30 years of experience. The patients considered in this study were all adults aged between 18 and 85 years. All cases were primary surgeries, not revisions. The surgical approach used was canal wall-up (CWU).

#### 2.4.1. Manual Dissection Procedure

Cholesteatoma was removed using fine microsurgical instruments under a high-magnification microscope. Care was taken to avoid applying excessive mechanical force and to preserve the integrity of the ossicular chain. The procedure was considered complete upon visual confirmation of a clean incus surface by the surgeon.

#### 2.4.2. CADISS-Assisted Dissection Procedure

**Figure 1:**
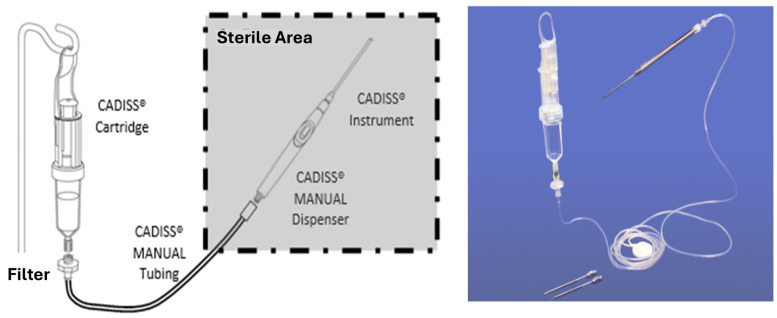
The CADISS^®^ System kit used in this study.

Mesna (5%) was delivered through a sterile, disposable kit supplied by the manufacturer, which includes a cartridge pre-filled with Mesna solution, a cartridge-to-handpiece connection tube with an integrated filter, and a manual pump handpiece, as shown in Figure 1.

The solution was applied directly onto the incus and surrounding structures for a maximum of 4 min. The entire middle ear cavity was potentially exposed to the solution; however, based on extensive clinical usage in ENT and other surgical fields, no adverse effects on the inner ear or neurotoxicity have been reported in the literature, nor in our series. The mechanism of action relies on Mesna’s ability to cleave disulfide bonds in keratinized matrix components, facilitating dissection along natural cleavage planes. The estimated cost of the CADISS kit ranges between EUR 90 and EUR 120 per procedure. The endpoint criteria were identical to the manual dissection group: visual confirmation of a clean surface without residual pathology [21,22].

### 2.5. Sample Processing for VP-SEM

Immediately after retrieval, samples were fixed in 2.5% glutaraldehyde (in 0.1 M phosphate buffer, pH 7.4) for 48 h. Post-fixation included two phosphate buffer rinses (10 min each), 1 h exposure to 1% osmium tetroxide, tannic acid impregnation (1% in distilled water, 30 min), and final rinses in distilled water. Samples were dried on absorbent paper, mounted on aluminum stubs with carbon tape, and directly imaged using a Hitachi SU3500 VP-SEM (Hitachi, Japan) at 30 Pa pressure and 6 kV acceleration. This imaging protocol, previously validated by our group, preserved tissue hydration and avoided dehydration-related artifacts. Images were obtained using secondary electron mode (SE/UVD) and backscattered electron mode (BSE), with denser bone appearing bright and residual soft tissue appearing dark, thus allowing for surface area quantification [23,24,25].

### 2.6. Qualitative Analysis

**Figure 2:**
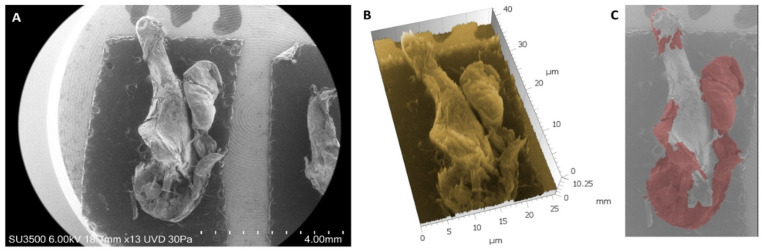
Qualitative evaluation at low magnification of a sample treated with a conventional surgical technique: VP-SEM (**A**) magnification 13×; (**B**) 3D reconstruction; (**C**) the false color image emphasizes the presence of cholesteatoma residues.

Each sample was first analyzed at 13× magnification to detect any gross residual cholesteatoma (defined as >1 mm × 1 mm) before proceeding with quantitative measurements. The presence/absence of cholesteatoma debris with dimensions larger than 1 mm × 1 mm on the incus surface was recorded; an example is given in Figure 2.

### 2.7. Quantitative Image Analysis

**Figure 3:**
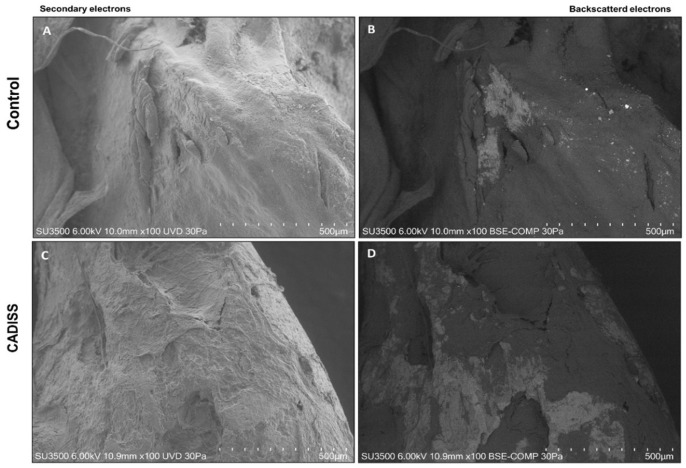
(**A**,**B**) The sample was treated with conventional surgical technique (control VP-SEM): (**A**) magnification 100× secondary electrons and (**B**) 100× backscattered electrons; the white area is indicative of exposed bone surface. (**C**,**D**) The sample was treated with the CADDIS technique (CADISS VP-SEM): (**C**) magnification 100× secondary electrons and (**D**) 100× backscattered electrons; the white area is indicative of exposed bone surface. Note the larger area compared with the control sample.

**Figure 4:**
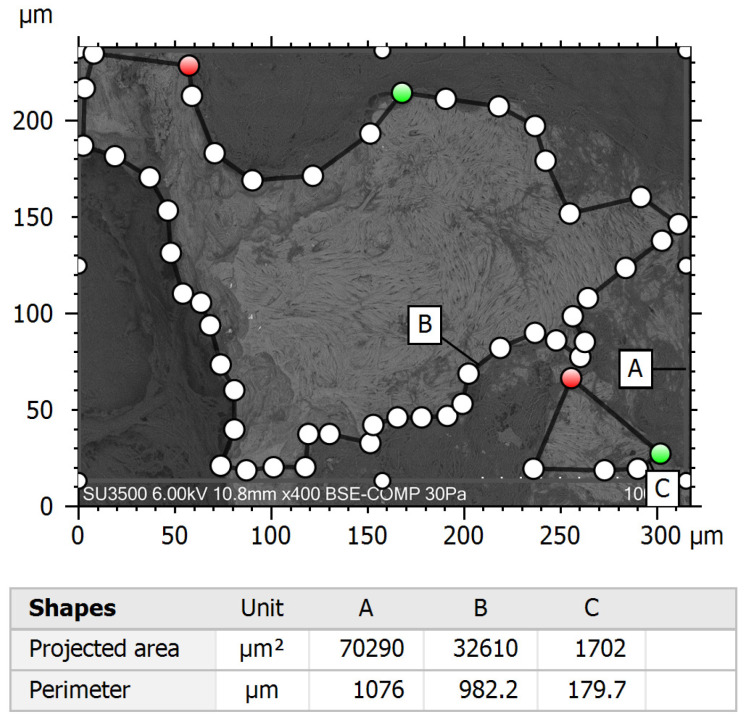
Area measurement results automatically acquired using the Hitachi Mountains Map software on BSE mode images.

Quantitative assessment of tissue removal was conducted using Hitachi Mountains Map 3D Advanced 8.2 software. Samples were randomly divided and assigned to two different investigators for analysis. Image analysis was performed on 10 images per sample, which were acquired at 100× magnification in the BSE mode (Figure 3) using the Area Selection software tool (Figure 4). The following parameters were measured using the software: total area (µm^2^), which means the entire surface area of the incus bone fragment, and clean area (µm^2^), the portion of the sample surface free of residual tissues. The process was blinded, with two independent investigators analyzing the images without knowledge of sample group assignments. Inter-rater reliability was assessed using the intra-class correlation coefficient (ICC) to confirm consistency in measurements between observers; it resulted in >0.75, suggesting good agreement. For each picture, the clean area/total area ratio, a normalized measure of cleaning efficiency, was calculated. This parameter was used for statistical analysis.

### 2.8. Statistical Analysis

Descriptive statistics were computed for all variables. The Shapiro–Wilk test was used to assess the normality of data distributions. As the data were not normally distributed, comparisons between the CADISS and manual groups were conducted using the Mann–Whitney U test. To assess the effect size, Cliff’s Delta was calculated as a robust, non-parametric estimator of group differences. This metric is particularly appropriate when distributions are not normal, or variances are unequal [26]. A significant threshold of *p* < 0.05 was applied.

### 2.9. Radiological Follow-Up

At one month postoperatively, all patients underwent diffusion-weighted MRI to evaluate the presence of residual cholesteatoma. These imaging results were used to validate the ultrastructural cleaning data and to assess early recurrence rates between the two surgical groups [21,22].

## 3. Results

### 3.1. Macroscopic and Qualitative Findings

The qualitative examination of incus surfaces under low-magnification VP-SEM (13×) provided immediate insight into the effectiveness of tissue removal techniques. In the manual dissection group, a substantial proportion of samples, 27 out of 35 (77%), exhibited residual cholesteatoma deposits exceeding 1 mm^2^. By contrast, in the CADISS-assisted group, only 3 out of 32 samples (9%) demonstrated comparable macroscopic remnants. These preliminary morphological observations underscore a striking disparity in surface cleanliness, favoring the chemically assisted dissection approach.

### 3.2. Quantitative Surface Analysis

To quantify this observed difference, images at 100× magnification were analyzed from each group: 350 images in the manual dissection group and 320 images in the CADISS group. Using BSE-mode VP-SEM imaging and digital image analysis software Hitachi Map 3D, both the cleaned surface area (sample surface without cholesteatoma residues) and the total surface area were measured for each sample.

**Figure 5:**
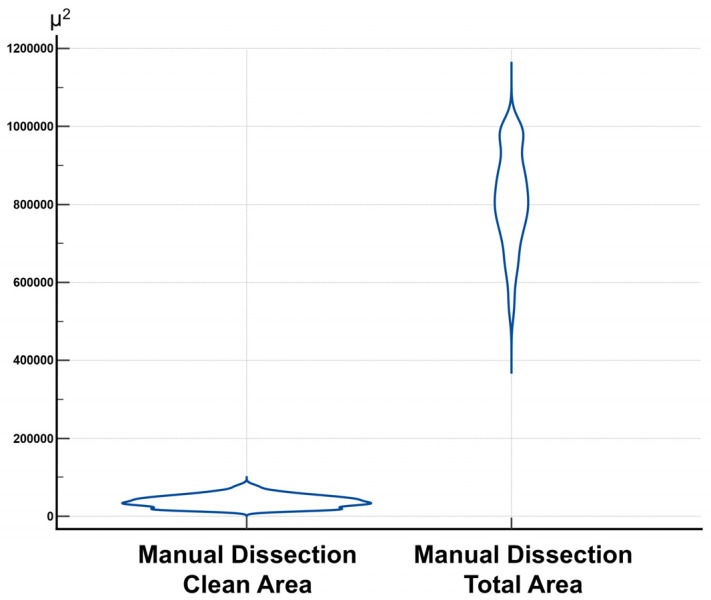
Distribution of clean area and total area values in the manual dissection group.

In the manual dissection group, the mean cleaned area was 38,311.18 µm^2^ (95% CI: 36,671.56–39,950.80), with a standard deviation (SD) of 15,596.25 µm^2^. The mean total area was 814,325.77 µm^2^ (95% CI: 801,462.70–827,188.84). The corresponding distributions for these parameters are graphically illustrated in Figure 5, and detailed descriptive statistics are presented in Table 1.

**Figure 6:**
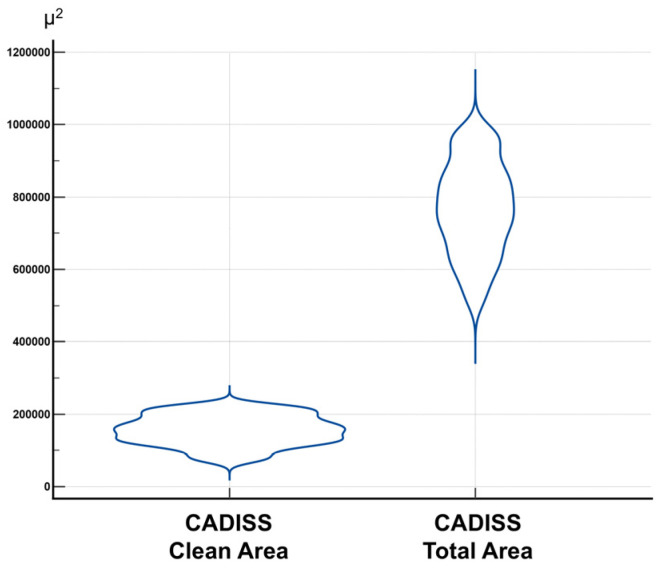
Distribution of clean and total area values in the CADISS-assisted group.

In the CADISS-assisted group, the mean cleaned area was 155,441.69 µm^2^ (95% CI: 150,898.13–159,985.23), and the mean total area was slightly lower at 768,308.05 µm^2^ (95% CI: 754,318.31–782,297.80). Distribution plots are provided in Figure 6, and the full dataset is summarized in Table 2.

### 3.3. Clean Area/Total Area Ratio and Statistical Distribution

**Figure 7:**
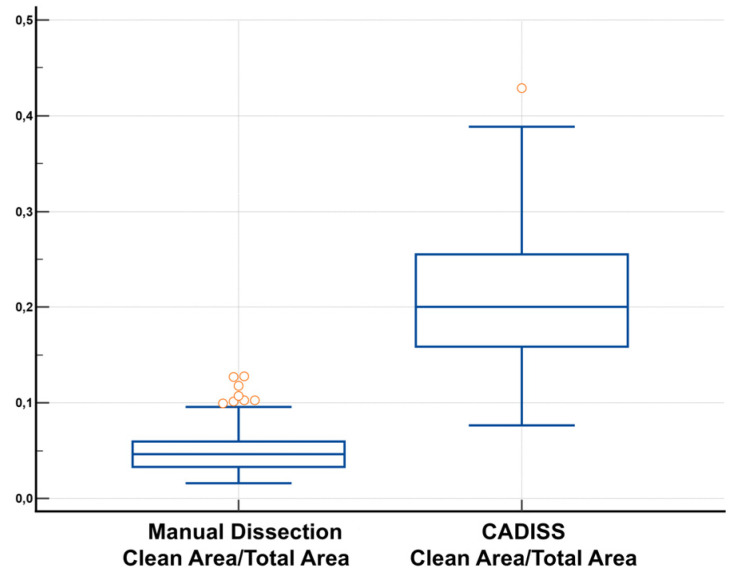
Box plot comparing clean area/total area ratios. Orange circles are outliers.

To allow for normalized intergroup comparisons, a clean area/total area ratio was computed for each image. This metric revealed a mean ratio of 0.0478 (SD = 0.0204) in the manual dissection group, with a range from 0.0161 to 0.1277. In contrast, the CADISS group demonstrated a markedly higher mean ratio of 0.2095 (SD = 0.0709), with values ranging from 0.0765 to 0.4284. These distributions are illustrated in Figure 7, while group-specific statistical metrics are presented in Table 3.

Before statistical testing, the normality of the data was assessed using the Shapiro–Wilk test, which confirmed non-normal distributions in both groups (manual dissection: W = 0.9563, *p* < 0.0001; CADISS: W = 0.9788, *p* = 0.0001). Consequently, a non-parametric approach was selected for hypothesis testing.

### 3.4. Comparative Statistical Evaluation

Given the non-Gaussian nature of the data, an intergroup comparison of the clean area/total area ratio was performed using the Mann–Whitney U test, a robust rank-based test for independent samples. The analysis revealed a statistically significant advantage in favor of the CADISS-assisted technique (U = 288.00, Z = −22.262, *p* < 0.0001). The medians were 0.04635 (manual group) and 0.2004 (CADISS group), with narrow 95% confidence intervals, confirming both the strength and precision of this difference. Full statistical outputs are provided in Table 4.

To contextualize the magnitude of the observed difference, Cliff’s Delta was calculated as a non-parametric effect size estimator. The result, −0.995, indicates an extremely large effect, suggesting that a randomly selected sample from the CADISS group will almost always demonstrate a higher cleaned surface ratio than that from the manual dissection group [19].

### 3.5. Considerations on Sample Size Adequacy

Although the total number of patients (67 patients: 35 in the manual dissection group and 32 in the CADISS group) may appear modest, several factors contribute to the statistical robustness and adequacy of the sample size. We carried out a high number of observations per group; each incus sample was analyzed through 10 high-resolution VP-SEM images, resulting in a total of 670 independent image-based measurements (350 manual, 320 CADISS). This provides substantial statistical power for detecting differences in morphometric outcomes.

Our results showed a strong effect size, and the difference between groups was not only statistically significant (*p* < 0.0001 by Mann–Whitney U test) but also associated with an extremely large effect size (Cliff’s Delta = −0.995). This indicates that the groups are almost entirely non-overlapping, and the observed difference is highly meaningful.

Our results presented narrow confidence intervals; the 95% confidence intervals for the clean area/total area ratios and other parameters were close, indicating precision in the estimates despite the sample size. We focused on the clean area/total area ratio as a normalized, reproducible metric for tissue removal efficiency. While other metrics (e.g., inflammatory response and operative time) may also be of interest, they were beyond the scope of this imaging-focused study.

We used appropriate non-parametric statistics, and the non-normal data distribution indicates that the non-parametric tests (Mann–Whitney U and Cliff’s Delta) were correctly employed. These methods are robust and require fewer assumptions, thus enabling reliable inference from smaller samples.

Finally, to ensure the homogeneity of surgical technique and operator, all surgeries were performed by the same senior surgeon, Prof Maurizio Barbara, using a CWU approach. This eliminated inter-operator and inter-procedural variability, increasing the internal validity and statistical efficiency of the study.

### 3.6. Radiological Validation

To validate the surgical and histological findings, radiological follow-up was performed using diffusion-weighted MRI at one month postoperatively (see Table 5). Among the 35 patients treated with manual dissection, 4 (11.4%) showed residual cholesteatoma signs. In the CADISS group (n = 32), only one patient (3.1%) demonstrated residual disease. These imaging results provide convergent evidence of CADISS’s efficacy in reducing early recurrence and suggest improved long-term disease control. The correlation between imaging and morphological data supports CADISS as a potentially superior technique for achieving surgical completeness [21,22].

## 4. Discussion

Recurrence in cholesteatoma is a multifaceted problem that can arise from various predisposing and procedural factors. One of the most significant contributors to recurrence is the presence of residual cholesteatoma after surgical intervention. Studies have emphasized that the incomplete removal of the disease matrix, particularly in complex cases, is a leading cause of regrowth [27,28]. Additionally, the type and infiltrative nature of the cholesteatoma matrix significantly contribute to recurrence risk, with certain types proving more resistant to complete surgical eradication [29].

The surgical techniques employed during the initial procedure are another pivotal factor. The choice between canal wall-up versus canal wall-down techniques can affect recurrence rates, with canal wall-down techniques generally yielding lower recurrence due to the more aggressive removal of all affected tissues [30,31]. However, the meticulousness of the surgeon plays a critical role, as inadequate visualization or exposure during surgery can increase the likelihood of leaving residual tissue behind, further complicating the outcome [32].

The presence of biofilms on residual cholesteatoma can protect bacteria against both host immune responses and antibiotics, adding another layer of difficulty in managing recurrences [33,34]. Moreover, the ongoing interactions of cholesteatoma with the surrounding tissues, including chronic inflammation and repeated infections, can exacerbate the persistence and regrowth of the disease [35]. Studies suggest that recurrent bouts of chronic infection not only contribute to disease persistence but can also create a cycle where the treatment effectively addresses symptoms temporarily while underlying pathology continues to thrive [35].

Improvements in surgical techniques are needed to further enhance cholesteatoma residue removal and lower recurrence rates. The CADISS^®^ system is based on the intraoperative application of Mesna (sodium 2-mercaptoethanesulfonate), a thiol compound with strong mucolytic and reductive properties. Chemically, Mesna acts by cleaving disulfide bonds (-S–S-) within extracellular matrix proteins, particularly those rich in cysteine residues such as keratin. Disulfide bridges are a key structural element in maintaining the integrity and adhesion of keratinized tissues, including the cholesteatoma matrix. By reducing these bonds, Mesna weakens the molecular cohesion of pathological tissue layers and facilitates cleavage at the natural dissection planes between the cholesteatoma matrix and healthy bone surfaces [36].

This selective action preserves adjacent structures and reduces the need for mechanical manipulation, which is particularly important when operating near vulnerable anatomical elements such as the facial nerve, stapes, or lateral semicircular canal. Furthermore, the localized application of Mesna minimizes systemic absorption, and studies have shown no significant ototoxicity or neurotoxicity when used in the middle ear. The CADISS system ensures standardized, controlled delivery of Mesna through a closed, sterile cartridge–handpiece unit, optimizing safety and reproducibility across surgical cases. The combination of chemical and mechanical action enables a more complete and atraumatic removal of residual cholesteatoma, especially in anatomically challenging regions.

### 4.1. Key Mechanisms of CADISS^®^ System in Enhancing Cholesteatoma Surgery

Mesna facilitates tissue dissection at natural cleavage planes, it acts by breaking disulfide bonds in keratinized and fibrotic tissue. In cholesteatoma surgery, this helps in loosening the cholesteatoma matrix from ossicular surfaces and bone and reducing adherence to critical structures such as the facial nerve, dura, or lateral semicircular canal.

The use of the CADISS^®^ system reduces mechanical trauma, whereas the traditional manual dissection can cause microfractures of ossicles, as well as damage to the facial nerve, labyrinth, or sigmoid sinus. The CADISS^®^ system softens the pathological tissue, enabling atraumatic removal with minimal mechanical force, thus reducing the risk of iatrogenic injury.

Surgical completeness is enhanced, as the residual matrix on the incus or in deep recesses (e.g., sinus tympani) is the main cause of recurrence, and CADISS improves both the visibility and accessibility of these areas. The ability to achieve complete matrix clearance was evidenced by higher clean area/total area ratios in our VP-SEM study.

The use of the CADISS^®^ system enables a safer single-stage surgery. By improving precision, CADISS may support single-stage procedures (e.g., tympanoplasty + ossiculoplasty) and avoidance of planned second-look surgeries in selected cases. This reduces the overall surgical burden for the patient.

Safe and controlled application is also an advantage of the CADISS^®^ system. Mesna is delivered via a closed, disposable handpiece system, and its application is localized, limiting systemic exposure. No significant ototoxicity or neurotoxicity has been reported in middle ear application.

Our imaging and clinical data demonstrated a statistically significant improvement in surface cleaning (*p* < 0.0001, Cliff’s Delta = −0.995) and lower MRI-detected residual disease (3.1% vs. 11.4%). These findings validate CADISS as a tool that translates into better surgical and clinical outcomes.

This study offers compelling evidence that CADISS-assisted dissection yields significantly superior outcomes compared to conventional manual techniques in the removal of the cholesteatoma matrix from ossicular bone surfaces. Quantitative analysis revealed a markedly higher clean area/total area ratio in the CADISS group, with statistical testing via the Mann–Whitney U test confirming this difference to be highly significant (*p* < 0.0001). Moreover, the effect size, measured by Cliff’s Delta (−0.995), denotes an extremely large difference, underscoring the clinical relevance of this technique [26].

### 4.2. Radiological Validation and Clinical Relevance

Postoperative diffusion-weighted magnetic resonance imaging (DW-MRI) is now widely regarded as a reliable tool for detecting residual disease following cholesteatoma surgery [21,22]. In our study, one-month postoperative imaging showed a substantially lower residual rate in the CADISS group (3.1%) compared to the manual dissection group (11.4%). These findings align with recent studies advocating early non-EPI DW-MRI for the detection of microscopic disease remnants.

Radiological findings complement the ultrastructural results obtained via VP-SEM and reinforce the value of CADISS in enhancing surgical completeness and potentially reducing recurrence, which are the key goals in the long-term management of cholesteatoma.

### 4.3. Mechanistic Insight and Histological Perspective

Previous studies have demonstrated that Mesna facilitates dissection by cleaving disulfide bonds within keratinized cholesteatoma tissue, thus enabling selective separation from underlying bone [13]. This mechanism is particularly advantageous in anatomically constrained areas, like the sinus tympani or facial recess. Reducing the need for mechanical force also minimizes trauma to the surrounding healthy structures, including the ossicles [13,15].

Our study extends these findings by providing quantitative ultrastructural evidence through VP-SEM imaging. Unlike dehydrated SEM or traditional histology, VP-SEM preserves tissue hydration and offers accurate visualization of soft tissue residues [15]. The significantly cleaner bone surfaces observed in the CADISS group validate its efficacy.

### 4.4. Implications for Surgical Practice

The complete eradication of the cholesteatoma matrix is essential, as residual epithelium is the main cause of recurrence [1,8]. Achieving this in deep or hidden recesses remains a challenge. CADISS improves dissection precision in these areas and may enhance outcomes, particularly when paired with endoscopic access [11,12,21].

Recent large-scale analyses have also contributed new insights into pediatric cholesteatoma management. Miller et al. [36] evaluated outcomes in over 6000 pediatric cases across different surgical modalities. They found that tympanoplasty (TM) and TM with canal wall-down mastoidectomy (TM-CWD) resulted in fewer total procedures and lower hearing aid dependency than TM with canal wall-up mastoidectomy (TM-CWU), without significant differences in speech development. These findings support a tailored, outcome-based approach to cholesteatoma surgery, especially in complex or recurrent cases.

Preserving ossicular integrity is also critical for postoperative hearing. Manual dissection can increase the risk of ossicular chain trauma or microfractures. In contrast, CADISS may improve functional outcomes by reducing mechanical stress [14,16].

### 4.5. Study Strengths and Limitations

This study’s strengths include its objective, reproducible metrics (clean area/total area ratio) and the use of Cliff’s Delta as a robust effect size estimator [26]. The integration of VP-SEM imaging offers high-resolution insights into bone–matrix interactions [23].

Limitations include a short (1-month) follow-up period. While early results are promising, longer-term data are needed to assess recurrence, audiological recovery, and patient-reported outcomes [36,37,38,39,40,41,42,43,44]. Additionally, this study did not evaluate operative time, which could be modestly affected by CADISS use.

### 4.6. Future Directions and Research Outlook

Future studies should validate these findings in prospective, controlled trials that assess recurrence over time, hearing outcomes, and surgical duration. Combining CADISS with endoscopic approaches may improve visualization and accessibility in complex regions.

Innovative directions may include optimizing Mesna formulations or integrating real-time detection systems, such as fluorescent-tagged probes or enzyme-activated markers to identify residual matrix intraoperatively.

## 5. Conclusions

This study provides robust quantitative and morphological evidence supporting the superior efficacy of CADISS-assisted dissection in cholesteatoma surgery. Compared to traditional manual techniques, the use of CADISS significantly improved the removal of the cholesteatoma matrix from ossicular bone surfaces, as demonstrated by both VP-SEM-based ultrastructural imaging and a markedly higher clean area/total area ratio. The statistical robustness of these findings, confirmed by a *p*-value < 0.0001 and an extremely large effect size (Cliff’s Delta = −0.995), underscores the clinical relevance of this technique.

Importantly, radiological follow-up with diffusion-weighted MRI further corroborated the morphological results, revealing a lower incidence of residual disease in patients treated with CADISS. These converging lines of evidence suggest that chemically assisted dissection not only improves surgical precision but may also contribute to reduced recurrence risk and better preservation of ossicular structures, which are the key goals of middle ear surgery.

While further prospective, in vivo studies are warranted to evaluate long-term outcomes and operational considerations such as surgical duration, the current data strongly support the integration of CADISS into standard cholesteatoma surgical protocols. Its adoption has the potential to enhance disease eradication, reduce the burden of revision surgery, and ultimately improve functional and prognostic outcomes for patients.

## Figures and Tables

**Table 1:** Descriptive statistics for the manual dissection group.

Manual Dissection Clean Area		Manual Dissection Total Area	
Sample size	350	Sample size	350
Lowest value	159,840,000	Lowest value	5,288,240,000
Highest value	840,050,000	Highest value	9,926,240,000
Arithmetic mean	383,111,829	Arithmetic mean	8,143,257,686
95% CI for the Arithmetic mean	366,715,635 to 399,508,022	95% CI for the Arithmetic mean	8,014,627,013 to 8,271,888,359
Standard deviation	155,962,530	Standard deviation	1,223,550,150
Shapiro–Wilk test	W = 0.9642 reject normality (*p* < 0.0001)	Shapiro–Wilk test	W = 0.9622 reject normality (*p* < 0.0001)

**Table 2:** Descriptive statistics for the CADISS-assisted group.

CADISS Clean Area		CADISS Total Area	
Sample size	320	Sample size	320
Lowest value	740,350,000	Lowest value	5,121,330,000
Highest value	2,217,450,000	Highest value	9,723,100,000
Arithmetic mean	1,554,416,875	Arithmetic mean	7,683,080,531
95% CI for the Arithmetic mean	1,508,981,353 to 1,599,852,397	95% CI for the Arithmetic mean	7,543,183,101 to 7,822,977,962
Standard deviation	413,115,583	Standard deviation	1,271,996,139
Shapiro–Wilk test	W = 0.9678	Shapiro–Wilk test	W = 0.9691

**Table 3:** Descriptive statistics for clean area/total area ratios.

Manual Dissection Clean Area/Total Area		CADISS Clean Area/Total Area	
Sample size	350	Sample size	320
Lowest value	0.01610	Lowest value	0.07650
Highest value	0.1277	Highest value	0.4284
Arithmetic mean	0.04782	Arithmetic mean	0.2095
95% CI for the Arithmetic mean	0.04567 to 0.04997	95% CI for the Arithmetic mean	0.2017 to 0.2173
Standard deviation	0.02044	Standard deviation	0.07092
Shapiro–Wilk test	W = 0.9563 reject normality (*p* < 0.0001)	Shapiro–Wilk test	W = 0.9788 reject normality (*p* = 0.0001)

**Table 4:** Mann–Whitney U test results.

	Manual Dissection Clean Area/Total Area	CADISS Clean Area/Total Area
Sample size	350	320
Lowest value	0.01610	0.07650
Highest value	0.1277	0.4284
Median	0.04635	0.2004
95% CI for the median	0.04297 to 0.04898	0.1927 to 0.2107
Interquartile range	0.03300 to 0.05960	0.1587 to 0.2551
Hodges–Lehmann median difference	0.1546	
95% Confidence interval	0.1464 to 0.1627	
Mann–Whitney test (independent samples)		
Average rank of the first group	1,763,229	
Average rank of the second group	5,096,000	
Mann–Whitney U	28,800	
Test statistic Z	−22,262	
Two-tailed probability	*p* < 0.0001	

**Table 5:** MRI-assessed residual cholesteatoma at one-month follow-up.

	Patients	Positive MRI 1 Month After Surgery
Manual dissection	35	4 (11.4%)
CADISS	32	1 (3.1%)
Total	67	5 (7.4%)

## Data Availability

Images captured by SEM are stored in our server and are available upon request.
